# Proton Beam Therapy for Non-Small Cell Lung Cancer: Current Clinical Evidence and Future Directions

**DOI:** 10.3390/cancers7030831

**Published:** 2015-07-02

**Authors:** Abigail T. Berman, Sara St. James, Ramesh Rengan

**Affiliations:** 1 Department of Radiation Oncology, University of Pennsylvania, Philadelphia, PA 19104, USA; 2 Department of Radiation Oncology, University of Washington Medical Center, Seattle, WA 98195, USA; E-Mails: sstjames@uw.edu (S.S.J.); rengan@uw.edu (R.R.)

**Keywords:** lung cancer, radiotherapy, re-irradiation, proton beam therapy (PBT), post-operative radiation therapy (PORT)

## Abstract

Lung cancer is the leading cancer cause of death in the United States. Radiotherapy is an essential component of the definitive treatment of early-stage and locally-advanced lung cancer, and the palliative treatment of metastatic lung cancer. Proton beam therapy (PBT), through its characteristic Bragg peak, has the potential to decrease the toxicity of radiotherapy, and, subsequently improve the therapeutic ratio. Herein, we provide a primer on the physics of proton beam therapy for lung cancer, present the existing data in early-stage and locally-advanced non-small cell lung cancer (NSCLC), as well as in special situations such as re-irradiation and post-operative radiation therapy. We then present the technical challenges, such as anatomic changes and motion management, and future directions for PBT in lung cancer, including pencil beam scanning.

## 1. Introduction

Although proton beam therapy (PBT) was initially conceived in 1930, only recently has this technology become readily available for the treatment of cancer patients [1]. Proton beam therapy, through its characteristic Bragg peak, has the potential to decrease the toxicity of radiotherapy, and, subsequently improve patient outcomes. The initial success of PBT was largely in prostate cancer [2] and pediatric malignancies [3]; however, as the number of proton centers has increased and the technology has advanced, PBT has been incorporated into the treatment paradigm of many types of malignancies.

Lung cancer is the leading cancer cause of death in the United States with over 158,000 estimated deaths in 2015 [4]. Radiotherapy is an essential component of the definitive treatment of early-stage and locally-advanced, and the palliative treatment of metastatic lung cancer. When treating to definitive doses, the toxicity of radiotherapy for lung cancer can be significant, with esophagitis [5] as a dose-limiting toxicity in the acute setting and pulmonary complications of treatment-related pneumonitis and fibrosis in the subacute and late period [6]. Therefore, PBT for lung cancer holds great potential to decrease toxicity by eliminating dose delivered to the surrounding critical structures and thereby can improve the therapeutic ratio. Herein, we present the existing data in early-stage and locally-advanced non-small cell lung cancer (NSCLC), as well as in special situations such as re-irradiation and post-operative radiation therapy. We then present the technical challenges, and future directions for PBT in lung cancer.

## 2. A Primer on Proton Beam Therapy Physics

**Figure 1:**
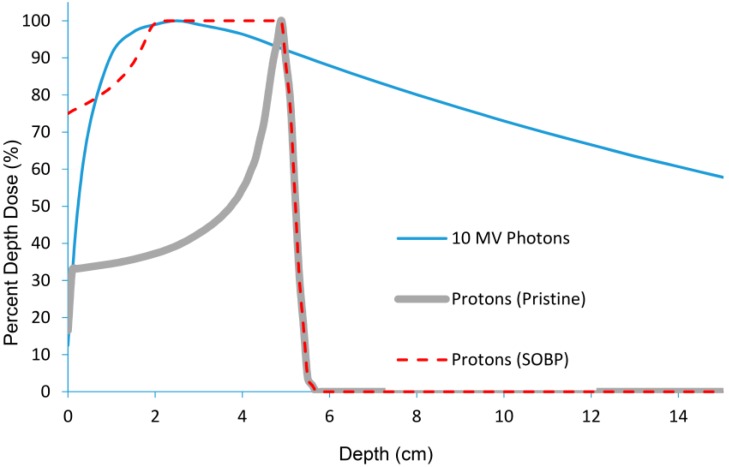
Percent depth dose curve of proton beam *vs.* photon beam demonstrating that protons do not deposit dose beyond the prescribed depth, whereas photon do.

The defining physical characteristic of proton depth-dose curves is the peak at the end of the proton range where most of the energy from the protons is deposited, beyond which the dose is negligible. This characteristic peak is known as the Bragg Peak and is a signature of all heavy charged particles. The advantage of proton radiation therapy is that the total dose (also known as the integral dose) deposited in patients treated with protons is generally less than the dose deposited in patients treated with high energy photons (that do not have a sharp decrease of dose). The depth of the Bragg Peak is dependent on the energy of the protons. By modulating the energy of the protons, it is possible to achieve a spread out Bragg Peak (SOBP). The maximum energy of the protons determines the distal range of the SOBP and the modulation of the energy of the protons determines the width of the SOBP. For comparison, curves showing the depth of dose for a pristine proton beam, a modulated proton beam (SOBP) and standard energy (10 MV) photons are shown in Figure 1.

In proton therapy, the beams are shaped laterally using either apertures, or by magnetically scanning a proton beam across the patient in the case of pencil beam scanning. This, combined with the ability to select the range in the patient allows for proton beams to be shaped in three dimensions.

In proton therapy, the dose is prescribed in units of cobalt Gray equivalent (CGE). This is the physical dose (the energy deposited per unit mass) multiplied by the relative biological effectiveness (RBE). When the physical dose is kept constant and the radiation quality (particle, energy) is changed, the biologic and clinical effect is different. RBE is used to relate the biological effect to a reference radiation (^60^Co). For external beam radiation using photons and electrons, the RBE is unity. For proton therapy, a RBE of 1.1 is used [7].

One of the challenges using proton beam therapy is that, unlike in external beam photon radiation, the uncertainties associated with the dose deposition are not uniform in all directions. Protons are exquisitely sensitive to the electron density of the material through which they pass and an additional uncertainty has to be accounted for in proton radiation therapy plans. In lung cancer, where the difference in electron density between the soft tissue of the chest and mediastinum and the lung is significant, range uncertainty due to uncertainties in the electron density is an important consideration. To ensure adequate coverage of the target, accounting for range uncertainties results in the high dose region in proton therapy plans to be less conformal to the target than in photon therapy. The sharp distal fall off of the proton dose results in the low dose region being more conformal than what may be achieved using high energy photons and, in many cases, achieves the goal of sparing organs at risk.

## 3. Proton Beam Therapy for Locally-Advanced Non-Small Cell Lung Cancer

The most common treatment paradigm for a medically fit, good performance status patient with locally-advanced non-small cell lung cancer is definitive concurrent chemoradiation [8]. Over the past decade, the introduction of 3-D conformal radiotherapy has reduced the toxicity of radiation delivered with older techniques [9], and intensity modulated radiation therapy (IMRT) further improve dose delivery with photons with some studies reporting a decrease in clinically significant treatment-related pneumonitis from 32% to 8% 10. Early dosimetric studies demonstrated that proton beam therapy is able to further reduce the dose to critical structures including the lungs, esophagus, heart, spinal cord, and brachial plexus [10]. Of note, PBT has been shown to decrease all the lung dosimetric parameters that have been shown to be predictive of radiation pneumonitis including V_5_, V_20_, and mean lung dose.

**Figure 2:**
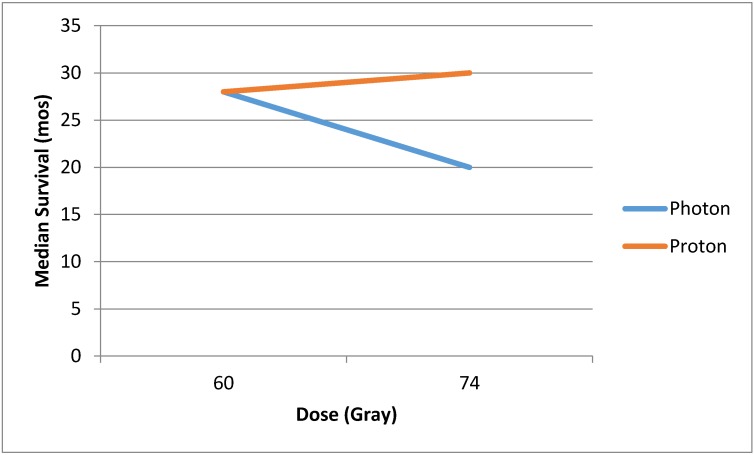
Hypothetical benefit of dose escalation with proton beam therapy over photon radiotherapy, as demonstrated as detrimental in Radiation Therapy Oncology Group (RTOG) 0617.

In addition to decreasing toxicity, PBT holds the hope of improving local control by allowing for the safe delivery of higher radiotherapy dose. Locoregional failure after concurrent chemoradiation can be as high as 50% [11,12], and locoregional control (LRC) has been correlated with long-term survival [13]. The role of radiotherapy dose escalation to improve local control is controversial: some reports have shown higher biologically effective dose of radiation to correlate with improved survival [14], but others have shown that dose escalation may be detrimental. For example, in Radiation Therapy Oncology Group (RTOG) 9410, in addition to testing the question of concurrent *vs.* sequential chemotherapy, the investigators tested the question of dose escalation and found that the 69.6 Gy arm was worse than the 60 Gy arm [8]. RTOG 0617, a randomized trial of standard dose (60 Gy) *vs.* high-dose (74 Gy) radiotherapy, was closed early in June 2011 when a planned interim analysis showed that the high-dose arms had worse survival than the standard-dose arms. This study recently reported a median overall survival was 28.7 months for standard-dose *vs.* 20.3 months for high-dose radiotherapy, which was statistically significant. They found that there were more treatment-related deaths in the high-dose arm (eight *vs.* three patients), and there was more severe esophagitis (21% *vs*. 7%). There was a suggestion on multivariable analysis that heart V5 and V30 were important predictors of overall survival [15]. Therefore, dose escalation may only decrease survival by increasing treatment-related deaths due to normal tissue injury. Proton beam therapy has the promise of delivering dose-escalated RT [16], and improving locoregional control and overall survival, but without inducing treatment-related deaths (Figure 2).

There are prospective studies that have reported the clinical outcomes of proton beam therapy for locally-advanced NSCLC. MD Anderson Cancer Center reported their 44-patient phase II study of chemoradiation with 74 Gy and carboplatin/paclitaxel chemotherapy. The treatment was well-tolerated with no Grade 4 or 5 proton-related adverse events. There was one patient with Grade 3 radiation pneumonitis. Local recurrence was 20.5%; overall survival was 86% at 1 year with a median survival of 29.4 months [17]. The University of Tsukuba first reported on 57 patients (24 stage IIIA and 33 IIIB) who received PBT without concurrent chemotherapy. Doses ranged from 50 to 84.5 Gy and treatment was completed in 89%). Overall survival period was 21.3 months, which compares favorably to other studies given that no concurrent chemotherapy was administered. Local control at 2 years was 64.1%. The same authors also reported their experience using combined modality PBT with 15 locally-advanced patients who received monthly cisplatin and vinorelbine, 74 Gy RBE for the primary site and 66 Gy RBE to the nodes, demonstrating minimal toxicity and a median survival of 26.7 months [18,19]. Similarly, the University of Florida reported their experience with 19 locally-advanced NSCLC patients treated with concurrent chemoradiation with carbo/taxol to a median proton dose of 74 Gy RBE; they found reasonable toxicity rates with only five patients with ≥Grade 3 toxicity [20].

The question of whether proton beam therapy is able to improve overall survival is being tested in a randomized phase III trial, RTOG 1308. A total of 560 inoperable Stage II-IIIB NSCLC patients are being randomized to photon *vs.* proton chemoradiotherapy; the dose delivered is 70 Gy using either modality, with the option to decrease to as low as 60 Gy if the dose constraints to the organs at risk cannot be met [21]. The authors set 28 months (close to the median survival in the standard-dose arm of RTOG 0617) as the median survival time as the primary objective.

## 4. Proton Beam Therapy for Early-Stage Non-Small Cell Lung Cancer

While stereotactic radiation therapy for early-stage NSCLC yields outstanding local control and low toxicity rates, proton beam therapy has the potential to improve the therapeutic ratio even further. Proton therapy, due to its lack of low-dose exit bath, has the potential to decrease the rate of secondary malignancies in a population who may live long enough to realize these effects.

Initial dosimetric comparisons have shown that PBT can be advantageous in the treatment of early-stage NSCLC over 3D-CRT in reducing doses to the lung, heart, esophagus, and spinal cord [22,23,24].

Several series have reported on the clinical outcomes of proton beam therapy for early-stage NSCLC [25]. The University of Tsukuba initially reported on their phase I dose escalation study of hypofractionated PBT for early-stage lung cancer. They treated three patients with 50 Gy in 10 fractions and then dose escalated to 60 Gy in 10 fractions in 18 patients. They found reasonable 2-year local progression-free and disease-free survival of 95% and 79%, and no Grade 3 or higher toxicity [26] Subsequently, they reported on 55 medically-inoperable patients with stage I NSCLC treated with proton beam therapy using a dose of 66 Gy in 10 fractions to peripheral tumors and 72.6 Gy in 22 fractions to centrally-located tumors. The authors found a 2-year local control, progression-free survival, and overall survival of 97%, 88.7%, and 97.8%, respectively, with a Grade 3 pneumonitis rate of 3.6% [27]. Loma Linda reported their dose-escalation phase II study of hypofractionated PBT for early-stage NSCLC using a fractionation scheme of 51 Gy in 10 fractions in the first 22 patients and then 60 Gy in 10 fractions to the subsequent 46 patients. Overall, the 3-year local control and disease-specific survival were 74 and 72%, respectively [28]. The authors recently updated this experience, and they included a further dose escalation cohort, with a total dose of 70 Gy, still administered in 10 fractions. The 4-year overall survival was 18%, 32%, and 51%, in the 51, 60, and 70 Gy cohorts, respectively. There were no cases of ≥Grade 3 radiation pneumonitis. Of note, larger tumors had increased local recurrence and decreased survival [29].

Despite these reasonable clinical outcomes, there are dosimetric data demonstrating the limitations of proton beam therapy for SBRT due to range uncertainties. In one study of 10 patients with early-stage NSCLC, they compared proton plans to photon plans and found that the protons generate larger high-dose regions because of range uncertainties, but smaller low-dose regions [30]. Therefore, critical structures adjacent to the target could receive a higher dose with proton therapy than photon-based SBRT. This same group has improved upon the technique and has shown that passive scatter proton therapy delivered as an arc improves the conformality of the large high-dose region created by a 3D-proton plan and improves organ-at-risk dose, such as the lung and chest wall. They generated proton plans using arcs with passively scattered proton therapy and intensity modulated proton therapy (IMPT). They found that IMPT-Arc had the most conformal dose distributions and the lowest low-dose lung values [31]. There continue to be barriers to the implementation of proton arc therapy, including creating the mechanical gantry and couch to be able to deliver the therapy in a short overall treatment time. While not currently in use clinically, proton arc holds the promise to improve upon photon-based stereotactic body radiation, an already highly-effective and low-toxicity treatment.

## 5. Special Situations for Proton Beam Therapy

### 5.1. Re-Irradiation

Recurrent lung cancer limited to the thorax poses a challenging clinical problem; the majority of patients, approximately 60%, will have already had radiotherapy once to the chest. Surgery and/or radiation can offer patients a second chance for a cure, however, both carry high risk for morbidity and mortality in such a heavily pre-treated population. PBT is one potential solution for re-irradiation for recurrent NSCLC as it is able to spare previously radiated tissue that lies beyond the target [32]. The University of Pennsylvania has reported the results of 24 NSCLC patients treated on a prospective study of PBT for re-irradiation for recurrent tumors. Patients were stratified into low-volume clinical target volume (≤250 cc) or high-volume (>250 cc) and treated to a median dose of 66.6 Gy (36–74), with 54% receiving concurrent chemotherapy. They found that there were two deaths in the high-volume cohort that were possible related to re-irradiation, and therefore additional exclusion criteria (effusion, prior radiation toxicity) have been added to the ongoing prospective study [33]. McAvoy *et al.* reported on the re-irradiation of 102 patients to a median re-irradiation dose of 60 Gy. Esophageal and pulmonary toxicity (≥Grade 3) occurred in 7% and 10%, respectively. They identified that higher T stage, squamous histology, poor performance status, and larger re-irradiation targets to be poor prognostic factors for survival [34]. Therefore, proton beam therapy often offers the possibility of radiation for patients in whom photon-based treatment would be relatively contraindicated, and further studies are warranted to determine the ideal selection criteria for this aggressive local therapy.

### 5.2. Post-Operative Radiation Therapy for Locally-Advanced NSCLC

Post-operative radiation therapy (PORT) for non-small cell lung cancer (NSCLC) is routinely given in the setting of mediastinal nodal (N2) disease or positive margins. Much of the controversy regarding whether or not PORT is beneficial in the setting of N2 disease is centered on the risk/benefit ratio of radiotherapy. In older studies, such as the PORT meta-analysis [35], there was no benefit to PORT in N2 disease identified. However, this was thought to be due to older, more toxic radiation technique [36], and newer analyses have demonstrated the benefit to PORT in this setting [37,38]. Therefore, proton beam therapy and its possibility to decrease the toxicity of radiation, has a great potential in PORT where the therapeutic window is particularly small. The benefit of proton therapy, in particular intensity modulated proton therapy, has been shown dosimetrically [39], and has been shown by the University of Pennsylvania in an early analysis to provide excellent clinical outcomes with minimal toxicity [40].

## 6. Caution Using Proton Beam Therapy

### 6.1. Anatomic Changes

**Figure 3:**
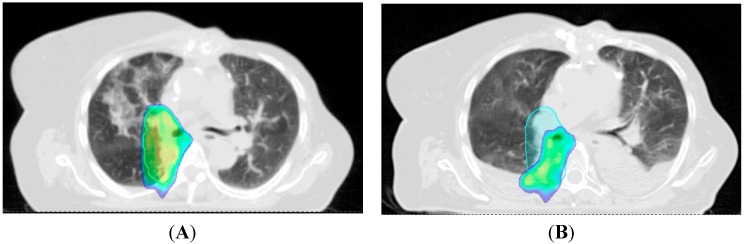
Example Showing the Sensitivity of Proton Beam Therapy to Anatomic Changes. Patient being treated with pencil beam scanning for a cT3N0 NSCLC developed an effusion after two weeks of radiotherapy (panel **A**: dose colorwash at initial simulation; panel **B**: dose colorwash at verification scan 2 weeks into radiotherapy). Planning tumor volume outlined in light blue. Dose colorwash shown cutoff at 90% in both panels. In panel **B**, compared to panel **A** (dose distribution at initial CT simulation), the target volume is significantly undercovered.

Given the sensitivity of proton beam therapy to anatomic changes in the thorax, adaptive re-planning is often needed to adjust the dose distribution while a patient is receiving radiotherapy. In particular, the lung cancer patient is at a high-risk for anatomic changes that can develop quickly, such as pleural and pericardial effusions or atelectasis. In the MD Anderson phase II trial, they found that nine of the 44 patients required modifications to their original treatment plans after repeat scans during treatment showed compromised target coverage or exceeding constraints to the organs-at-risk. They found that adaptive planning was used more often for large tumors that exhibited significant volume reduction during treatment [41]. They found that adaptive re-planning improved target coverage, where it would have been compromised in two patients, and sparing of organs-at-risk, including the esophagus and spinal cord. An example of adaptive re-planning due to change of dose distribution from the development of a pleural effusion mid-radiation is shown in Figure 3.

### 6.2. Motion Management

As proton beam therapy is exquisitely sensitive to anatomic changes, accounting for intrafraction and interfraction tumor motion is a key component of treating NSCLC with PBT. Such changes can be seen due to target motion from breathing, which is a significant issue in lung cancer, or due to setup uncertainties [42]. Respiratory motion quantification and motion mitigation strategies should therefore be strongly considered in all proton therapy lung patients. This can be performed by accounting for the target local during all phases of the breathing cycle, captured by a 4D CT simulation where the target is imaged cross-sectionally during approximately 10 phases of the breathing cycle.

All patients undergoing proton therapy for NSCLC should undergo 4D CT simulation, where the tumor is observed on 8–10 phases of the breathing cycle. The primary treatment planning must take place on one scan series, and different institutions propose to account for motion in different ways. Wang *et al.* in evaluating different strategies for PBT therapy in lung cancer based on 4D CT scans, found that planning PBT was most robust when done so on the maximum intensity projection of end inhale, middle exhale, and end exhale images [43]. Alternatively, radiation can be delivered only during a specific portion of the breathing cycle (such as deep inspiration breath hold or gated radiotherapy delivery).

## 7. The Future of Proton Beam Therapy Technology: Pencil Beam Scanning

Intensity modulated proton beam therapy (IMPT) as mentioned above has the potential to improve upon passively-scattered proton therapy, which is the modality which has been used in clinical care to-date. IMPT optimizes the energies and intensities of proton pencil beams to create a highly-conformal dose distribution. Implementation into the clinic is challenging because of complexity in treatment planning, motion management, and quality assurance. M.D. Anderson Cancer Center (Houston, TX, USA) reported on their series of 34 patients with thoracic cancers (NSCLC, small cell carcinoma, large cell neuroendocrine tumor, and other thoracic malignancies), all with minimal tumor motion (<5 mm during respiration), who had a significant dosimetric advantage with IMPT over standard proton delivery techniques [44]. They found that IMPT improved lung, heart, and esophageal dosimetric parameters over both IMRT and passive-scattered proton therapy. They were able to maintain the maximum deviation from the target dose coverage was <5%. Of note, adaptive re-planning was used in 27% of patients. Additionally, Kesarwala *et al.* reported that IMPT may allow for elective nodal irradiation without an increase in dose delivered to the surrounding critical organs when compared to IMRT [45].

## 8. Conclusions

The technical and clinical data for proton beam therapy for both early-stage and locally-advanced NSCLC is quickly maturing. The composite data demonstrates that PBT holds the potential to decrease toxicity, and, in doing so, improve clinical outcomes in lung cancer. Proton beam therapy delivery techniques continue to improve and many of the technical challenges of sensitivity to anatomic changes and motion are being addressed. In settings such as re-irradiation and post-operative radiation therapy, where the therapeutic window is particularly narrow, proton beam therapy holds a crucial role. While we await the results of randomized data, proton therapy may be considered as an option for select patients with NSCLC.
